# Declined expressing mRNA of beta-defensin 108 from epididymis is associated with decreased sperm motility in blue fox (*Vulpes lagopus*)

**DOI:** 10.1186/s12917-020-02697-6

**Published:** 2021-01-07

**Authors:** Ping Wu, Tao-lin Liu, Ling-ling Li, Zhi-ping Liu, Li-hong Tian, Zhi-jun Hou

**Affiliations:** College of Wildlife and Protected Area, Northeast Forestry University, Harbin, China

**Keywords:** *Vulpes lagopus*, epididymis, *vBD108*, asthenospermia, sperm motility

## Abstract

**Background:**

Fecundity is important for farm blue fox (*Vulpes lagopus*), who with asthenospermia have be a problem in some of farms in China. A key symptom of asthenospermia is decreased sperm motility. The decreased secreting beta-defensin108 (*vBD108*) of blue fox is speculated be related to asthenospermia. To clarify this idea, the mRNA expression of *vBD108* in testis and epididymis of blue foxes with asthenospermia were detected and compared to the healthy one. The antibody was prepared and analyzed by immunohistochemistry.

**Results:**

The *vBD108* in testis and epididymis was found both in blue fox with asthenospermia and healthy group by the method of immunohistochemistry. The expression of *vBD108* mRNA in testes (*P* < 0.05) and epididymal corpus (*P* < 0.0001) in asthenospermia group was lower than that in healthy group.

**Conclusions:**

These results suggested that *vBD108* deficiency may related to blue fox asthenospermia. Meanwhile, the study on the blue fox *vBD108* provides a hopeful direction to explore the pathogenesis of blue fox asthenospermia in the future.

## Background

β-defensins, well known cationic antimicrobial peptide rich in cysteine [1–4], belonging to a family of host-defense peptides, produced by multiple epithelial tissues and immune cells, played an important role in the innate immune response [5, 6]. Although most of the studies on β-defensins focused on their antibacterial and anti-tumor activities, there is growing evidence that β-defensins play a special role on motility of sperm in the epididymal of mammals [7, 8].

The addition of cauda epididymal fluid (CEF) of cattle containing β-defensin 126 (*BBD126*) can significantly improve the motility of bovine spermatozoa [9]. The results of some studies suggested that human β-defensin 1 (*hBD-1*) can affect the quality of sperm and improve sperm motility when exogenously added [10, 11]. Inhibition of the expression of *Bin1b*, a rat epididymis-specific β-defensin with antimicrobial activity [12], resulted in a decrease in the binding of *Bin1b* to sperm and a significant decrease in sperm motility and progressive movement [8]. The epididymal specific β-defensin 15 (*Defb15*) was down regulated in vivo, which would result significantly decreased the total motility and progressive motility of rat spermatozoa [7].

Some farm blue fox with low sperm motility was paid attention and it always be called asthenospermia [10]. As the β-defensin is involved in the acquisition of sperm motility, the asthenospermia of blue fox may be related to the decrease of β-defensin secretion was suspected. The object of this study is to confirm if the β-defensin 108, especially in testis and epididymis, is related to the sperm motility.

## Results

### Detection of *vBD108* protein in testis and epididymis

**Fig. 1:**
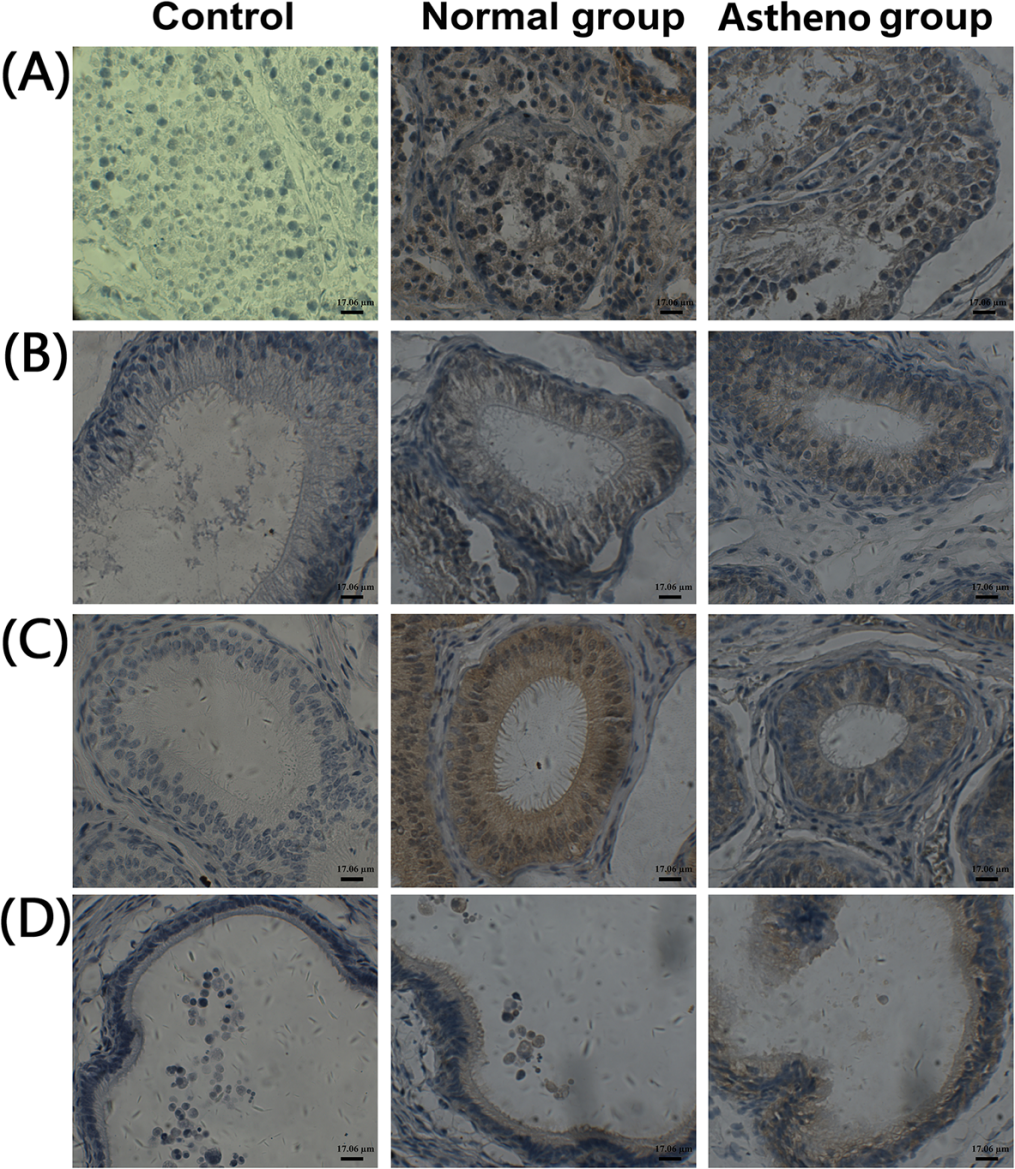
Immunohistochemical analysis of *vBD108* expression in testis and epididymis. Tissue sections from both healthy and asthenospermia blue fox were stained with anti-*vBD108* antibody except control group. Bar=17.06 µm. Representative images of tissue sections from *n*=3 blue foxes were shown. **a**: Testis; **b**: Caput; **c**: Corpus; **d**: Cauda

These animals did not use other drugs or other experiments before the experiment. In the immunohistochemical experiment, three healthy blue foxes (3/5) and three blue foxes with asthenospermia (3/5) were used. The reason is that about half of the samples were randomly selected for immunohistochemical detection. The results of immunohistochemical detection of *vBD108* were shown in Fig. 1. Staining of the testis and caput, corpus and cauda of the epididymis from the control group without primary antibody was negative. Brown coloration indicative of positive staining was found both in the testis, caput epididymis, corpus epididymis as well as in the cauda epididymis of the asthenospermia blue fox and healthy one compare to the control group.

### Detection of mRNA expression level of *vBD108* in testis and epididymis of asthenospermia and the healthy blue fox

**Fig. 2:**
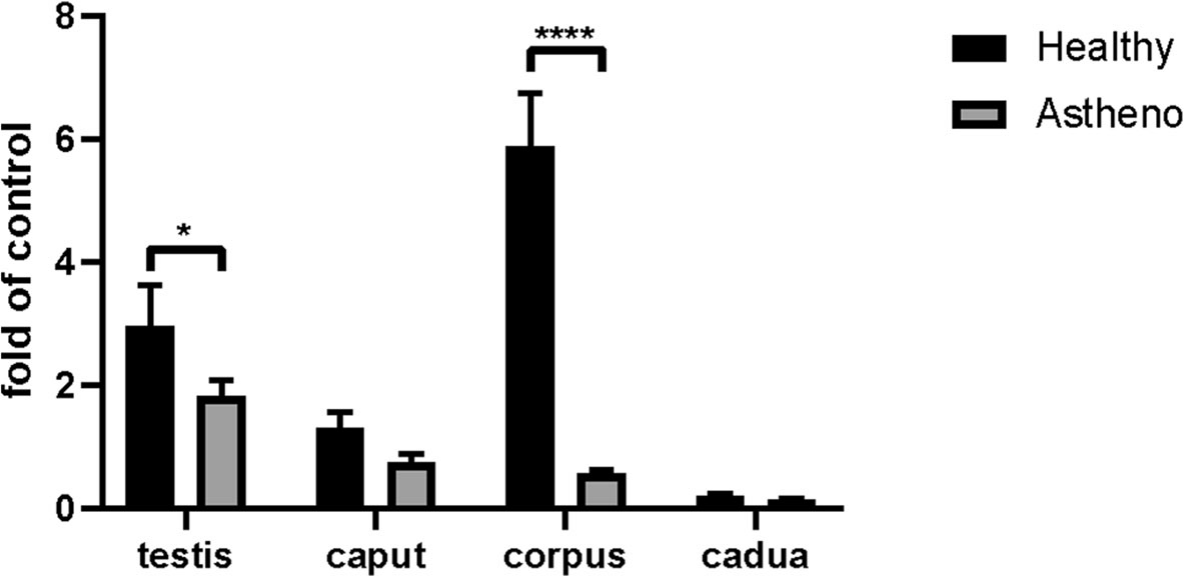
Relative expression of *vBD108* in testis and epididymis of asthenospermia and healthy blue fox. Data are expressed as the mean ± SD, *n*=3. The difference between averages is determined by using Sidak’s multiple comparisons test (*, *P* < 0.05; ****, *P* < 0.0001)

The results of immunohistochemistry showed that there was a difference in the expression of *vBD108* between healthy blue fox and asthenospermia blue fox. We used qPCR technique to quantitatively analyze the expression levels of *vBD108* in testes, and caput, corpus and cauda of epididymis of asthenospermia blue fox and the healthy one. The expression level of *vBD108* mRNA in testes and epididymis of blue fox was analyzed as shown in Fig. 2. We found that the relative expression of *vBD108* both in testes (*P* < 0.05) and corpus epididymis (*P* < 0.0001) of healthy blue fox was significantly higher than that of blue fox with asthenospermia. Among the healthy fox and asthenospertic one, the difference of *vBD108* in corpus of epididymis is especially apparently (*P* < 0.0001).

## Discussion

The epididymal lumen environment is an androgen response organ composed of specific ions, small organic molecules and proteins responsible for sperm maturation and storage [13]. In addition to β-defensin, androgen also plays an important role in several physiological processes of epididymis. Several studies have reported that androgens may regulate some β-defensins [14]. Two mouse β-defensins (*Defb41* and *Defb42*) are regulated by androgens by Q-RT-PCR and in situ hybridization [15]. Sperm-associated antigen 11A is expressed exclusively in the principal cells of the mouse caput epididymis in an androgen-dependent manner [16]. Human epididymis specifically expresses β-defensin 118 (*DEFB118*) (formerly *ESC42*) is regulated by androgen [17]. Six genes (*Defb18, 19, 20, 39, 41* and *42*) of mouse epididymal β-defensin were identified by bilateral orchiectomy and androgen supplementation, which were completely regulated by androgen [18]. Moreover, the time difference of seasonal expression is also the characteristic of β-defensin. The mRNA level of *SPAG11A* in the epididymis of wild ground squirrels in breeding season was significantly higher than that in non-breeding season [16]. A lot of studies have shown that the expression of β-defensin in testes and epididymis of different species (rat, mouse, sheep, goat) is time-dependent. The expression of sheep *Defb124* gene in sheep testes and epididymis and the goat beta-defensins 104a in the epididymis have age differences [19, 20]. Likewise, the expression of β-defensins (*Defb15* and *− 49*) in rat epididymis and *Defb41* in mouse testes are different during postnatal development [7, 15, 21]. To sum up, we speculate that *vBD108* may have the same differences in age distribution and androgen dependence and seasonal expression as these β-defensins. However, these functions of *vBD108* still need to be tested by experiments in the future.

A great number of studies have shown that β-defensin is involved in antimicrobial activity and regulating immune host [5, 22]. Loss of β-defensin gene expression can lead to asthenospermia, which is usually associated with low sperm motility [10, 23]. Sperm motility is considered to be one of the most important sperm functions affecting natural conception [24]. More and more studies have shown that mammalian β-defensin has an effect on sperm function. It is worth noting that after sperm are produced from the testis, they gain their motility and fertilization ability through the epididymis, which is called sperm maturation [13, 25]. Biology generally divides the epididymis into caput, corpus and cauda [9, 26, 27], which created unique and dynamic physiological environment provides conditions for sperm maturation [28–31]. A variety of region-specific epididymal β-defensins contribute to different microenvironments of epididymal sperm maturation in different segments were found [6]. The expression of *vBD108* protein in epididymis of blue fox with asthenospermia have an apparently difference compare with the healthy one, it suggests that *vBD108*, as a molecule of epididymal microenvironment, may be involved in the acquisition of sperm motility. This view is supported by some reports. A rat epididymal specific β-defensin (*Bin1b*) [12] can bind to sperm heads in different regions of the epididymis in different binding modes has been reported. Moreover, *Bin1b* is considered to be related to epididymal sperm maturation [8]. Human β-defensin 1 (*DEFB1*) interacts with chemokine receptor type 6 (*CCR6*) in spermatozoa, triggering Ca^2+^ mobilization, which is key for sperm motility [10]. β‑defensin *DEFB126*, a multifunctional glycoprotein, is adsorbed to the sperm surface during epididymal maturation [32]. All in all, these findings suggest that members of *vBD108* may be involved not only in antibacterial and host immunity, but also in the regulation of sperm motility.

Although our experimental results found that *vBD108* may be related to azoospermia in blue foxes, we need more experiments to determine the role of *vBD108* in sperm motility and male reproduction. We plan to clarify this problem through several aspects in following works. Firstly, *vBD108* antibody or *vBD108* protein are co-cultured with sperm to observe the changes of sperm motility. Secondly, the expression of *vBD108* gene in the epididymis of blue fox was knocked down by RNAi technique, and the changes of the binding ability of *vBD108* to sperm and the binding ability between sperm and ovum were analyzed. Finally, mating experiments were conducted to evaluate the changes in fertility of male blue fox with down-regulated *vBD108*.

## Conclusions

The results of this study demonstrate that low *vBD108* in corpus of epididymis was associated to asthenospermia and might be causal linked to low sperm motility. Meanwhile, the study on the blue fox *vBD108* provides a hopeful direction to explore the pathogenesis of blue fox asthenospermia in the future.

## Methods

### Animals and tissue collection

The five healthy and eight asthenospermia blue foxes are provided by Harbin Hualong Blue Fox breeding Co., Ltd. The age of the animals were two years old. The weight of the animals was from 17 to 22 kg. These experimental animals were all male. Wu et al. used sperm motility analysis system (IVOS) to detect sperm motility in fresh semen. Sperm forward motility is less than 50% or fast straight forward movement is less than 25%, which is called low sperm motility, also known as asthenospermia [33]. According to the International Animal Welfare Law, the blue fox was euthanized by intravenous injection of excessive pentobarbital sodium. The testes, and caput, corpus and cauda of epididymes were collected in April 2017 in College of Wildlife and Protected Area, Northeast Forestry University, Harbin, China. The samples were soaked in RNA preservation solution and stored at -80 °C. In addition, the tissue samples of each sample were cut into tissue blocks of 0.5 cm × 0.5 cm × 0.2 cm and fixed in 4% neutral formaldehyde fixation solution. Four SPF New Zealand rabbits were purchased from the Harbin Veterinary Research Institute, CAAS. The age of the rabbits ranged from 1 to 1.5 years and the weight of the animals were from 1.45 to 1.55 kg. Rabbits were fed with rabbit feed (SPF experimental rabbit feed) in 37 °C laboratory animal room. Two rabbits were kept in each cage and they had free access to water. The rabbits were euthanized after excessive intravenous injection of pentobarbital sodium at the end of the experiment.

### Preparation of polyclonal antibody against rabbit *vBD108*

A custom monoclonal antibody specific for *vBD108* was commercially generated by Genscript using a standard protocol. The synthesized *vBD108* protein was diluted with sterilized PBS (Takara, Dalian) buffer and emulsified with Freund Adjuvant (Takara, Dalian) at a volume ratio of 1:1, and then used for inoculation. 1 ml of immune antigen emulsion (0.25 mg /ml) in 200–300 µl per each site was injected into the abdominal cavity of rabbits. Operate in the same way every 14 to 16 days. Rabbits were immunized three times, the first one was immunized with Freund complete adjuvant, and the last two times, Freund incomplete adjuvant was selected [34]. After the third immunization, the blood was collected from the auricular vein on the 12th day and the serum was separated.

### Enzyme-linked immunosorbent assay

The titer of serum was determined by indirect ELISA [35] with respective pre-immunized serum as negative control. In short, the gradient diluted *vBD108* was used to coat the enzyme labeling plate transversely, and the gradient diluted rabbit *vBD108* positive serum and negative serum were added longitudinally to the enzyme labeling plate. The absorbance value was measured and the optional coating concentration of *vBD108* and the best working concentration of serum were determined. Then the antibody titer of *vBD108* was determined by the same method with the determined optimal antigen coating concentration. The ratio of OD490nm in positive serum to OD490nm in negative serum was between 1.5 and 3.0, which was the zero point of positive reaction, and the corresponding serum dilution was the antibody titer of serum.

### Immunohistochemical detection of *vBD108* in testis and epididymis of blue fox with asthenospermia and the healthy one

The samples of blue foxes with asthenospermia (*n*=3) and the healthy one (*n*=3) were used by immunohistochemistry. The fixed tissue samples were dehydrated with different concentrations of ethanol, the dehydrated tissues were treated with anhydrous ethanol and xylene 1:1 mixture for 30 min, and xylene solution 20 min for twice. After transparent operation, the tissue needs to be soaked in wax for 3 hours and embedded 3–5 µm thick slices, fixed on chromic acid-treated slides, spread them in a pool at 30 °C, bake at 37 °C overnight or 60 °C for 2 hours. After tissue dewaxing, the slides were heated in sodium citrate at 98 °C in 10 min for antigen repair, 3% H_2_O_2_ for 10 min, 5 min washing for 3 times, 1 × PBS for 5 minutes. Goat serum was diluted 10 times with 1 × PBS and sealed for one hour. The primary antibody was diluted according to 1:1000, each piece was 100 µl (wax circle), overnight at 4 °C (recovery of the primary antibody), and 5 min washed with 1 × PBS for three times after incubation.

The slides were incubated with the second antibody (HRP labeled goat anti-rabbit IgG) 37 °C for 1 hour with the diluent in the DAB chromogenic kit (Takara, Dalian). H_2_O_2_ was added to the diluted DAB chromogenic solution according to the ratio of 1:1000, and then added to the glass slide for color development. Re-dye with hematoxylin for one minute, add dilute hydrochloric acid to return to red, and then return to blue in light ammonia water. Finally, it was dehydrated, sealed with neutral gum and observed under microscope. Controls were subjected to the same procedure except without adding primary antibody.

### Acquisition of organizational cDNA

According to the operation instructions of Trizol Reagent kit (BioTeke, Beijing), the total mRNA, nucleic acid and protein analyzer NanoDrop-2000 (Beijing) was used to determine the concentration and purity of total RNA, and then the adjusted concentration was about 0.5 µg/ml. According to canine β-defensin 108 gene (accession: XM_003432123) in NCBI database, degenerate primer was designed using primer 5.0. The coding sequence of *vBD108* was amplified by the following primers: forward primer F: 5’- GAAGCCKTGTCTGCCTCTG − 3’ and reverse primer R: 5’- GATCATTCCTTGGGTGTAG − 3’. Reverse transcription was performed according to the instructions of HiScrip® II Q RT SuperMix for qPCR (+ gDNA wiper) kit (Vazyme, Nanjing). The first step was as follows: 20 µl total reaction system, including 4× gDNA wiper Mix 4 µl, 12 µl total RNA extracted from each tissue, 42 °C for 2 min. The second step: 5× HiScript II qRT SuperMix II 4µ l, the first step reaction solution 16 µl, reverse transcription conditions: 50 °C for 15 min, 85 °C for 5 sec, products were stored at -20 °C.

### qPCR

In order to detect the expression of *vBD108* in testis and epididymis of blue fox, the primers for qPCR detection of fox *vBD108* gene (F: 5’-CTCGCCCTGCTCTTCTTTCT-3’, R: 5’-CGTGGCCATTTAAACACCTC − 3’) were designed according to canine β-defensin 108 gene (accession: XM_003432123) in NCBI database. According to the reaction system and reaction conditions of AceQ qPCR SYBR Green Master Mix kit (Vazyme, Nanjing), real-time quantitative PCR reaction was carried out. The cDNA obtained in the experiment was used as a template, and the qPCR detection primers mentioned above were added to the 96-well plate (with repetitive holes) dedicated for fluorescent quantitative PCR, and the following reagents were added at one time. The upstream and downstream primers were respectively 1 µl, SYBR Green Master Mix (2×) 7.5 µl, cDNA solution 3 µl, ddH_2_O supplement to the final reaction volume of 16 µl. GAPDH was used as the internal reference, the sequence of it was amplified by the following primers: the forward primer F: 5’- AACATCATCCCTGCTTCCAC − 3’ and the reverse primer R: 5’- ATGCCTGCTTCACTACCTTCTT − 3’. The fluorescence quantitative results were all processed by 2^−△△CT^ method, and the relative expression of target gene was analyzed by GraphPad Prism Version 8.0.2.

### Statistical analysis

We performed statistical analysis using GraphPad Prism Version 8.0.2. Results are shown as mean ± standard deviation. Data correlation analysis was performed between groups. We considered results to be significant at *P* < 0.05.

## Data Availability

The datasets generated and/or analysed during the current study are available in the GenBank (https://www.ncbi.nlm.nih.gov/nuccore/XM_003432123) and the accession number is XM_003432123.

## Abbreviations

mRNA: Messenger ribonucleic acid.
CEF: Cauda epididymal fluid
qPCR: Real-time Quantitative PCR Detecting System
CCR6: Chemokine receptor type 6
RNA: Ribonucleic acid
SPF: Specific pathogen free
CAAS: Chinese Academy of Agricultural Sciences
H_2_O_2_: Hydrogen peroxide
PBS: Phosphate buffered solution
ELISA: Enzyme-linked immunosorbent assay
cDNA: Complementary deoxyribonucleic acid
GAPDH: Glyceraldehyde-3-phosphate dehydrogenase
SD: Standard deviation
